# EEG Channel Selection Based User Identification via Improved Flower Pollination Algorithm

**DOI:** 10.3390/s22062092

**Published:** 2022-03-08

**Authors:** Zaid Abdi Alkareem Alyasseri, Osama Ahmad Alomari, João P. Papa, Mohammed Azmi Al-Betar, Karrar Hameed Abdulkareem, Mazin Abed Mohammed, Seifedine Kadry, Orawit Thinnukool, Pattaraporn Khuwuthyakorn

**Affiliations:** 1 ECE Department, Faculty of Engineering, University of Kufa, Najaf 54001, Iraq; zaid.alyasseri@uokufa.edu.iq; 2 Information Technology Research and Development Center (ITRDC), University of Kufa, Najaf 54001, Iraq; 3 MLALP Research Group, University of Sharjah, Sharjah P.O. Box 27272, United Arab Emirates; oalomari@sharjah.ac.ae; 4 Department of Computing, UNESP—São Paulo State University, Bauru 19060-560, Brazil; papa.joaopaulo@gmail.com; 5 Artificial Intelligence Research Center (AIRC), College of Engineering and Information Technology, Ajman University, Ajman P.O. Box 20550, United Arab Emirates; mohbetar@bau.edu.jo; 6 Department of Information Technology, Al-Huson University College, Al-Balqa Applied University, Al-Huson, Irbid 21110, Jordan; 7 College of Agriculture, Al-Muthanna University, Samawah 66001, Iraq; Khak9784@mu.edu.iq; 8 College of Computer Science and Information Technology, University of Anbar, Ramadi 31001, Iraq; mazinalshujeary@uoanbar.edu.iq; 9 Department of Applied Data Science, Norrof University College, 4608 Kristiansand, Norway; seifedine.kadry@noroff.no; 10 College of Arts, Media, and Technology, Chiang Mai University, Chiang Mai 50200, Thailand; orawit.t@cmu.ac.th

**Keywords:** EEG, biometric, *β*-hill climbing, flower pollination algorithm, feature selection, auto-repressive

## Abstract

The electroencephalogram (EEG) introduced a massive potential for user identification. Several studies have shown that EEG provides unique features in addition to typical strength for spoofing attacks. EEG provides a graphic recording of the brain’s electrical activity that electrodes can capture on the scalp at different places. However, selecting which electrodes should be used is a challenging task. Such a subject is formulated as an electrode selection task that is tackled by optimization methods. In this work, a new approach to select the most representative electrodes is introduced. The proposed algorithm is a hybrid version of the Flower Pollination Algorithm and β-Hill Climbing optimizer called FPAβ-hc. The performance of the FPAβ-hc algorithm is evaluated using a standard EEG motor imagery dataset. The experimental results show that the FPAβ-hc can utilize less than half of the electrode numbers, achieving more accurate results than seven other methods.

## 1. Introduction

Over many years, our world has transferred into a digital community, where each subject lives with an unique digital identifier [1]. Indeed, there are many identifiers, such as identification passwords and cards. At the same time, these identifiers can be easily circumvented, stolen, and forgotten [2]. Therefore, personal characteristics or behaviors can be used to strengthen identification applications. Such techniques, so-called biometrics, use several in-person information to allow more robust identification systems, such as face and voice recognition, fingerprint information, and iris data, among others [3].

On the other hand, the widespread and influential deployment of biometric systems leads to a new challenge, which is called “spoofing” [1,4]. Such an attack is classified as the most dangerous in security systems since it is designed to break the biometrics systems’ security, thus allowing unwarranted persons to obtain admission to the system [2].

In real life, there have already been several spoofing attacks on biometrics systems, such as face spoofing (printed photos and 3D mask attacks [5,6]), fake fingerprints (gummy fingers), finger–vein systems fooled through a piece of paper [7], iris recognition systems fooled by an eyeball opposite to the scanner of iris, and voice recognition fooled by replaying a voice recording opposite to the recognition system speaker [7]. Therefore, people are looking for biometric authentication systems that can grant access to a person based on invisible characteristics, thus becoming harder to be attacked by an external threat. In this context, one shall refer to user authentication based on brain signals, which can be captured by the well-known electroencephalogram (EEG) exam [8].

The EEG is a clinical test that places electrodes on the person’s scalp to detect the brain’s electrical activities, which are further recorded for visualization purposes. Such information reflects the voltage currents inside the brain from ionic flows concerning the neurons’ activity [2]. Approaches to capture electrical brain signals can be categorized as invasive and non-invasive [9], where the former ones require surgery to embed electrodes in the brain. The electrocorticography brain–computer interface (ECoG BCI) is an example, which is usually intended for recording the movements of the arm [2]. Other signal types are used in the non-invasive approaches, such as functional magnetic resonance imaging and magnetoencephalography.

Many studies have proposed to solve issues relevant to identification in biometric applications. For instance, Jayarathne [10] gathered signals as a biometric approach from 21 test subjects to verify their identity. The authors employed the EMOTIV EEG Headset with 14 channels. The Common Spatial Patterns were used for feature extraction and Linear Discriminant Analysis for classification purposes. The proposed approach achieved a 96.97% recognition rate, which motivated the authors to claim that EEG signals might be an excellent approach to replace PINs when accessing ATMs. However, the selection of relevant channels that produces the optimal subset of EEG features is of prime importance for (i) reducing computational complexity, (ii) reducing over-fitting, and (iii) eliminating inconveniences during clinical application [11]. Table 1 presents some studies we thought might be relevant to EEG channel selection.

According to Table 1, many studies have worked on the channel selection problem with different methodologies such as Common Spatial Patterns, optimization, Pearson correlation coefficient, and additional connectivity metrics. Most existing works were implemented based on the data extracted from 64 EEG channels. Furthermore, most works reduced the number of channels and presented significant classification accuracy. In contrast, refs. [10,11] reported good classification performances, but the number of selected channels is still high. On the other hand, some studies have reduced channels up to 50%, but with a moderated classification rate.

Recently, several researchers proposed the use of optimization approaches to solve challenges with non-stationary signals [1,6,12,13]. In addition, EEG-based user identification with supervised classification and optimization methods has shown significant improvements compared to traditional techniques [2,14,15].

Signal acquisition is one of the significant problems concerning the EEG-based user identification technique, which is performed by placing electrodes on the head of a human [19,20,21]. In addition, such a process is usually uncomfortable since it requires good knowledge to place the sensors correctly. Additionally, some questions must be considered: “Is it essential to place all these electrodes on the head of persons?” and “Whether not, may we detect the most significant ones for user identification and then utilize fewer electrodes?”.

The above questions led our work to model the EEG channel selection as an optimization problem. Flower Pollination Algorithm (FPA) is a robust optimization method and has been successfully applied to many real-world problems [22]. Although FPA has proved to be a great success in finding optimal solutions to many issues, it suffers, like metaheuristic algorithms, from the inability to generate new solutions when it is stuck in local minima [23]. According to [1], the authors tested several meta-heuristic algorithms for EEG channel selection, with FPA achieving the most accurate results. However, it still has some problems, such as being stuck in local minima. For this reason, we propose to hybridize FPA with the local search optimizer β-hill climbing (β-hc) [24].

This work is one of the first to employ hybrid optimization methods with supervised classification methods for biometric user identification using EEG. The main point of hybridizing any two approaches is to complement their advantages and avoid their shortcomings. This work aims to learn the most critical EEG channels by proposing a hybrid approach composed of β-hc and FPA, named “FPAβ-hc”. Therefore, we expect to obtain more accurate results when applying optimization approaches to select optimal EEG channels. The main contributions of this work are summarized as follows:

1. To evaluate the proposed FPAβ-hc for EEG-based user identification. Such a hybrid approach aims to improve local pollination in FPA to avoid being stuck in local minima.
2. To perform an extensive study to select the most suitable classifier to guide the optimization process using FPAβ-hc. Our experiments showed that Support Vector Machines with Radial Basis Function (SVM-RBF) obtained the most effective results, thus being the preferred approach in this work.

The remainder of this article is organized as follows: Section 1 presents the main concepts regarding EEG signals, as well as related works about EEG-based identification. The proposed method is detailed in Section 2. The results are discussed in Section 3, the discussion is provided in Section 4, and the conclusions and future works are set out in Section 5.

## 2. Proposed Method

**Figure 1:**
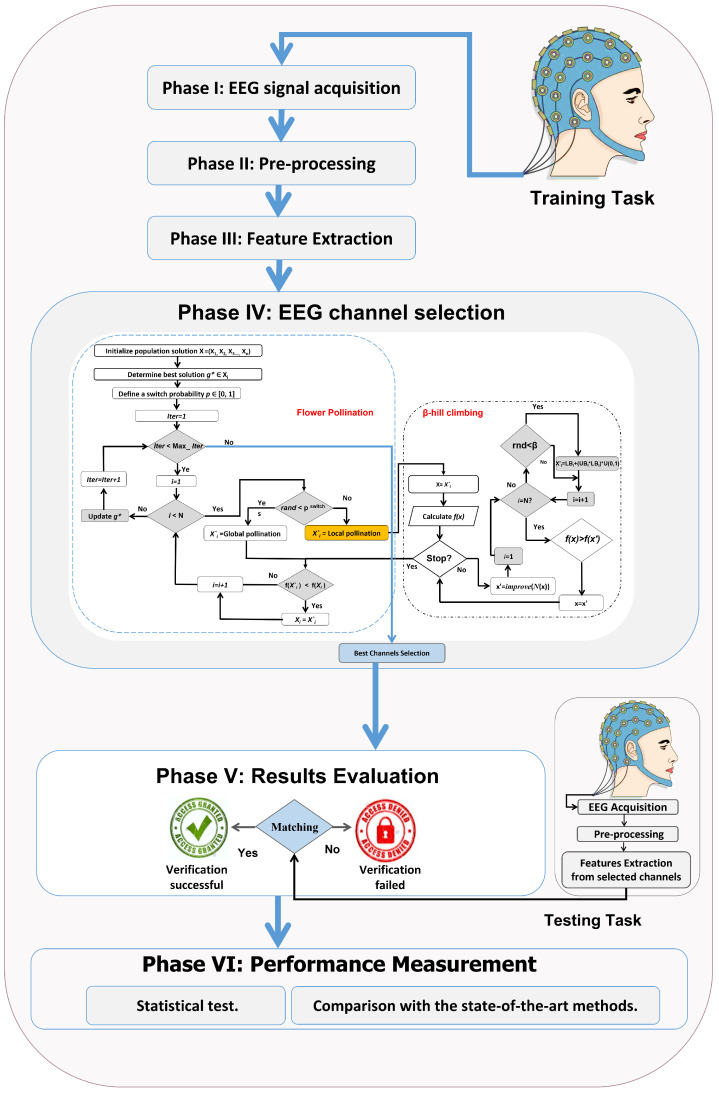
Proposed EEG-based user identification system using FPAβ-hc.

This section provides a detailed explanation of the proposed approach for EEG channel selection through FPAβ-hc, which comprises five steps such that every stage’s output acts as an input to the other one. The proposed approach is depicted in Figure 1.

### 2.1. EEG Signal Acquisition

**Figure 2:**
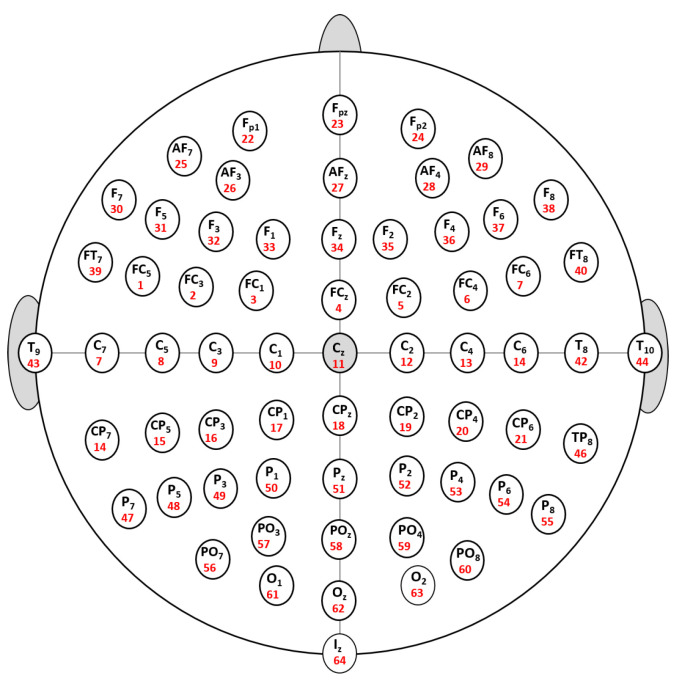
Distribution of the electrodes used in the study.

The EEG signal acquisition step is carried out over a typical EEG signal dataset. By using a brain–computer interface software called the BCI2000 system, the EEG signals are gathered from 109 healthy persons [25]. The EEG signals are acquired from 64 electrodes (i.e., channels), and 12 motor/imagery tasks are performed by every subject (i.e., 12 EEG signals records for each individual). Furthermore, AR features with three different numbers of coefficients are derived from these recordings: $AR_{5}$, $AR_{10}$, and $AR_{20}$. To reduce the dispersion of the EEG patterns and quickly process the extracted features, we compute the mean value of each electrode. The electrode distribution used in this work is depicted in Figure 2.

### 2.2. Pre-Processing

The original EEG signal is separated into six sub-signals, each comprising 10s. We utilized a notch filter, and a band-pass for denoising purposes for the EEG signal can be corrupted during recording.

### 2.3. Feature Extraction

Feature extraction plays a significant role in any authentication system. Therefore, the primary purpose of this step is to find unique information from the EEG signals. In this work, two feature extraction methods have been used to represent features: Wavelet Transform and Auto-regressive (AR) models. This work uses the Yule–Walker method to estimate the AR coefficients using the least-squares method with three different numbers of coefficients, i.e., 5, 10, and 20, as suggested by Rodrigues [1].

**Figure 3:**
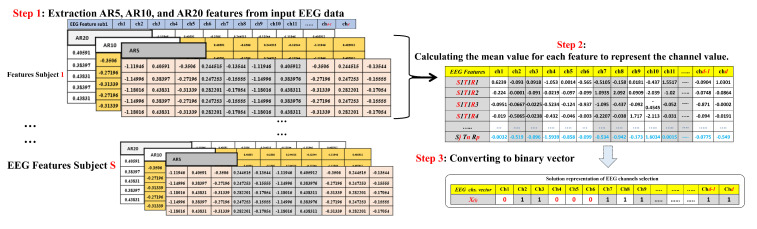
EEG feature representation.

First, we need to keep these features in a standard format of rows and columns. In general, the rows represent the AR feature values of each channel (electrode), and the columns represent the input EEG signals. Finally, a bidimensional matrix will be constructed. Figure 3 shows the final EEG dataset representation from data recorded from several subjects.

Notably, not all features are helpful for final decisions. Some of them will increase the complexity or will lead to achieving high miss-classification rates. We model the problem of selecting proper AR features as an optimization problem using the objective function defined in Section 2.4, where the channels that achieved the best results are the ones that will be selected.

### 2.4. Objective Function

**Figure 4:**
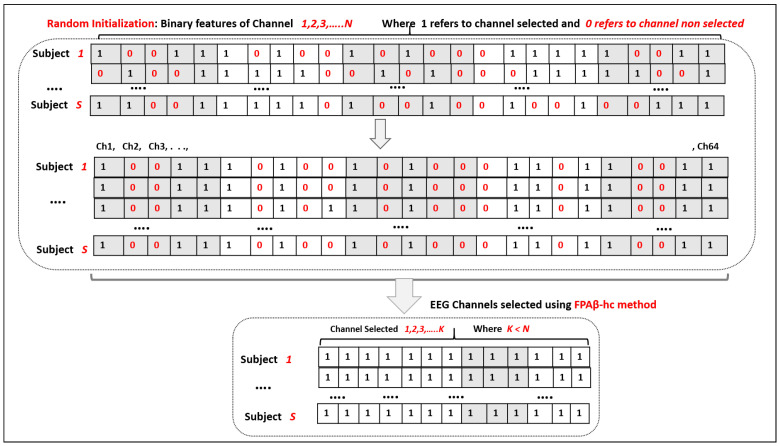
EEG channel selection using the proposed approach (FPAβ-hc).

Since the proposed method is initially designed to handle continuous-valued optimization problems, we need to map each possible solution onto a binary-valued position, for the EEG channel selection problem requires encoding each possible solution as a binary vector, where ‘0’ means the channel will not be selected and ‘1’ otherwise [1]. To restrict binary solutions only, we need to use the so-called “transfer function” *Z*, which refers to the *Z*-shaped transformation function. The primary purpose of using a transformation function is to convert a real-valued solution to another one with binary values suitable for the channel selection problem. This transformation function has been used in [1], where *Z* can be defined as follows:

(1) $$\begin{array}{c} Z\left(f\left(s_{i}^{t}\right)\right)=\left\{\begin{array}{cc} 1 & \phi >\sigma (f\left(s_{i}^{t}\right))\\ 0 & \mathrm{otherwise}, \end{array}\right. \end{array}$$

where

(2) $$\begin{array}{c} \sigma \left(f\left(s_{i}^{t}\right)\right)=\frac{1}{1+e^{-f(s_{i}^{t})}}, \end{array}$$

and ϕ∼U(0,1). Figure 4 illustrates how to build such a binary vector and to select the optimal EEG subset channels using the proposed FPAβ-hc.

Three steps must be considered when selecting the optimal subset of channels. First, a random initialization of the binary vector (representing the EEG channels) is conducted, where ‘1’ refers that a given channel will be selected, and ‘0’ denotes that the channel will not be selected. Second, the FPAβ-hc starts searching the space to find the optimal subset of channels, i.e., the one that can provide the highest accuracy rate based on Equation (3). Finally, we discard all channels with ‘0’ values and keep the remaining ones.

The objective function used to evaluate the classification performance of EEG channel selection is formulated below:

(3) $$Acc=\frac{TA+TR}{TA+FA+TR+FR}\times 100$$

where Acc denotes the objective function (accuracy rate) calculated for each row of the AR feature matrix, and TA, TR, FA, and FR represent the true acceptance, true reject, false acceptance, and false reject rates, respectively.

Algorithm 1 presents the proposed method that employs BFPAβhc for EEG-channel selection using the SVM-RBF classifier and Equations (1) and (2) as the transfer function.
**Algorithm 1** Hybridizing Flower Pollination Algorithm with β-hill climbing for EEG Channels Selection.1:**Input:**2:Initialize a population of *N* flowers/pollens with random solution3:Find the best solution $G_{sol}^{*}$ in the initial population4:Define a switch probability p∈[0,1]5:Channels = $\left\{Ch_{1},Ch_{2},\ldots ,Ch_{D}\right\}$6:**for***a* = 1 to *N* **do**7:   Evaluate fitness value of f(sol) based on 10-fold-CSV SVM and Accuracy rate of EEG Channels Selection [Equation (3)]8:**end for**9:Find $G_{sol}^{*}$, where $G_{sol}^{*}$∈ (1, 2, *…*, *N*)10:itr=011:**while**itr<Total_iterations**do**12:   **for** *j* = 1 to *N* **do**13:     **for** *i* = 1 to numberofchannels(D) **do**14:        **if** rnd≤p **then**15:          Draw a (d-dimensional) step vector ***L*** which obeys a Levy distribution16:          Global pollination via $sol_{i}^{itr}$ = $sol_{i}^{itr-1}$ + $L*(G_{sol}^{*}-sol_{i}^{itr-1})$17:          sigmoid($sol_{i}^{itr}$) = $\frac{1}{1+e^{-sol_{i}^{itr}}}$18:          **if** sigmoid($sol_{i}^{itr+1}$) >U(0,1) **then**19:             $sol_{i,j}^{\prime itr}=1$20:          **else**21:             $sol_{i,j}^{\prime itr}=0$22:          **end if**23:        **else**24:          Drawfromauniformdistribution∈[0,1]25:          Randomlychoosejandkamongallsolution26:          Dolocalpollinationvia $sol_{i}^{itr}$ = $sol_{i}^{itr-1}$ + ∈ ($sol_{j}^{itr}$ − $sol_{k}^{itr}$)27:          sigmoid($sol_{i}^{itr}$) = $\frac{1}{1+e^{-sol_{i}^{itr}}}$28:          **if** sigmoid($x_{i}^{itr}$) >U(0,1) **then**29:             $sol_{i,j}^{\prime itr}=1$30:          **else**31:             $sol_{i,j}^{\prime itr}=0$32:          **end if**33:        **end if**34:     **end for**35:     **Run β-hill climbing algorithm using $sol_{i,j}^{\prime itr}$**.36:     **while** Stop criterion is not met **do**37:        $New-sol_{i,j}^{\prime itr}$ = $N-Operator(sol_{i,j}^{\prime itr})$38:        $New-sol_{i,j}^{\prime \prime itr}$ = $\beta -Operator(New-sol_{i,j}^{\prime itr})$39:        **if** $f\left(New-sol_{i,j}^{\prime itr}\right)\leq f\left(New-sol_{i,j}^{\prime \prime itr}\right)$ **then**40:          replace $\left(New-sol_{i,j}^{\prime itr}\right)$ by $\left(New-sol_{i,j}^{\prime \prime itr}\right)$41:        **end if**42:     **end while**43:     $sol_{i,j}^{\prime itr}=New-sol_{i,j}^{\prime \prime itr}$44:   **end for**45:   Update $G_{sol}^{*}$, $where\;G_{sol}^{*}\in \left(1,2,\ldots ,N\right)$46:   itr=itr+147:**end while**48:**Output**49:Return $G_{sol}^{*}$: bestchannelssubsetwithhighestaccuracyrate.50:End

### 2.5. Experimental Setup

The dataset D is partitioned into three subgroups, $D=D_{1}\cup D_{2}\cup D_{3}$, which stand for the training, validation, and test sets, respectively. In addition, we decided to use 50% of the dataset for training purposes and 20% and 30% for the test and validation datasets, respectively, as suggested by Rodrigues et al. [1]. We adopted the classification accuracy of a Support Vector Machine with Radial Basis Function (SVM-RBF) over the validation set as the objective function to assist the optimization process. The main idea is to employ training and validation sets to find the subset of EEG features that maximize the classification accuracy over the latter. Finally, we select the best subgroups of channels that provide the highest accuracy rates and train the classifier once more. The final accuracy is assessed over the test set. The experiments have been performed utilizing a Lenovo Ideapad 310 PC, Intel Core-i7 with 2.59 Ghz processor, 8 GB of RAM, and Windows 10. The FPA and FPAβ-hc parameter values are presented in Table 2. Notice that *T* refers to the maximum number of iterations used in the experiments. These parameters have been selected based on our previous works [13,24].

## 3. Results

This section details the experiments used to evaluate the proposed approach; standard approaches in Section 3.1; comparison against standard FPA, β-hc, and FPAβ-hc in Section 3.2; and, finally, comparison with State-of-the-Art in Section 3.3.

### 3.1. EEG Classification Using Standard Machine Learning Approaches

Eight classifiers are selected from the literature according to their performance in EEG applications and evaluated their effectiveness to guide the optimization process proposed in the work: Linear Support Vector Machines, Artificial Neural Networks (ANN), Optimum-Path Forest (OPF), *k*-Nearest Neighbors (*k*-NN), Support Vector Machine with Radial Basis Function, Linear Discriminant Analysis (LDA), Decision Tree (J48), and Naive Bayes. Table 3 presents the parameter setting for these classifiers.

Since the proposed approach is non-deterministic, we computed the mean accuracy rate over 25 runs for avoiding bias outcomes as used in [1]. To evaluate the proposed approach, we considered five measures: (i) Accuracy (Acc), (ii) Number of selected channels (No. Ch), (iii) Sensitivity (Sen), (v) Specificity (Spe), and (iv) F-Score.

Table 4 presents the performance of FPAβ-hc with different classifiers. Regarding AR${}_{5}$ model, it has been observed that FPAβ-hc-SVM-RBF outperforms all methods on all evaluation measurements. The LDA stands out as the second-best approach with the five best values of Acc, Sen, Spe, and F-Score measures. Concerning $AR_{10}$ and $AR_{20}$, FPAβ-hc-SVM-RBF outperforms other methods for all evaluation metrics. Similar to its performance in $AR_{5}$, LDA stands out as the second-best performance method with the five best values for Acc, Sen, Spe, and F-Score in $AR_{10}$ and $AR_{20}$ models. One of the exciting details in Table 4 concerns that the proposed method (FPAβ-hc-SVM) achieved zero misclassification rates with the $AR_{20}$ model.

Furthermore, a statistical test is applied to determine whether there is a significant difference between FPAβ-hc-SVM-RBF and others. This study stated a null hypothesis as follows: “channel subset chosen by FPAβ-hc-SVM-RBF for user authentication is no better than channel subset chosen by FPAβ-hc with other classifiers”. The statistical test is based on the classification accuracy Acc obtained by all methods on the testing data. A t-test was conducted to test the hypothesis in this study, where the significance level was set to 0.05. As one can observe in Table 5, the p-value for all tests was below 0.05, which means the hypothesis is rejected. Accordingly, the channel subset obtained by FPAβ-hc-SVM-RBF is significantly better than FPAβ-hc with other classifiers, thus handling EEG-based person identification better.

### 3.2. Comparison against Standard FPA, β-hc, and FPAβ-hc

**Figure 5:**
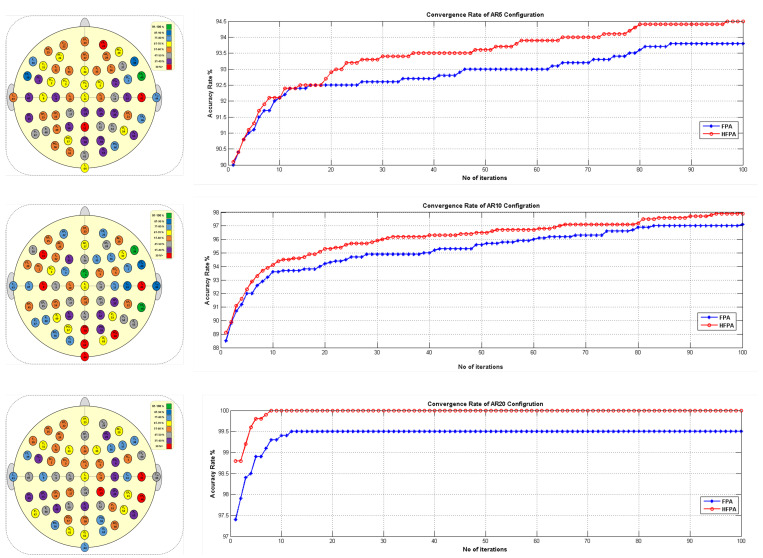
Convergence rate and the frequency of channel selection for FPAβ-hc and FPA.

After selecting the best classifier for the EEG-based person identification problem (i.e., SVM-RBF), standard FPA, β-hc, and FPAβ-hc are compared to select the best one for further comparison with some state-of-the-art techniques. Figure 5 depicts the convergence ratio, and the frequency of chosen electrodes for standard FPA and FPAβ-hc for 25 runs during the experimental evaluation using AR-5, AR-10, AR-20, and Wavelet Transform WT.

Table 6 presents the overall results concerning the comparison between standard FPA, β-hc, and FPAβ-hc. It is clear from the experimental outcomes that the proposed method considerably improves the results considering all auto-regressive coefficients. Regarding the number of selected channels, the proposed approach showed quite good performance. On the other hand, β-hc selected the lowest number of channels, but it yielded worse results than the proposed method concerning the remaining measures (i.e., Acc, Sen, Spe, and F-Score). Even though β-hc obtained the lowest number of channels, it has less classification accuracy when compared with FPAβ-hc.

**Figure 6:**
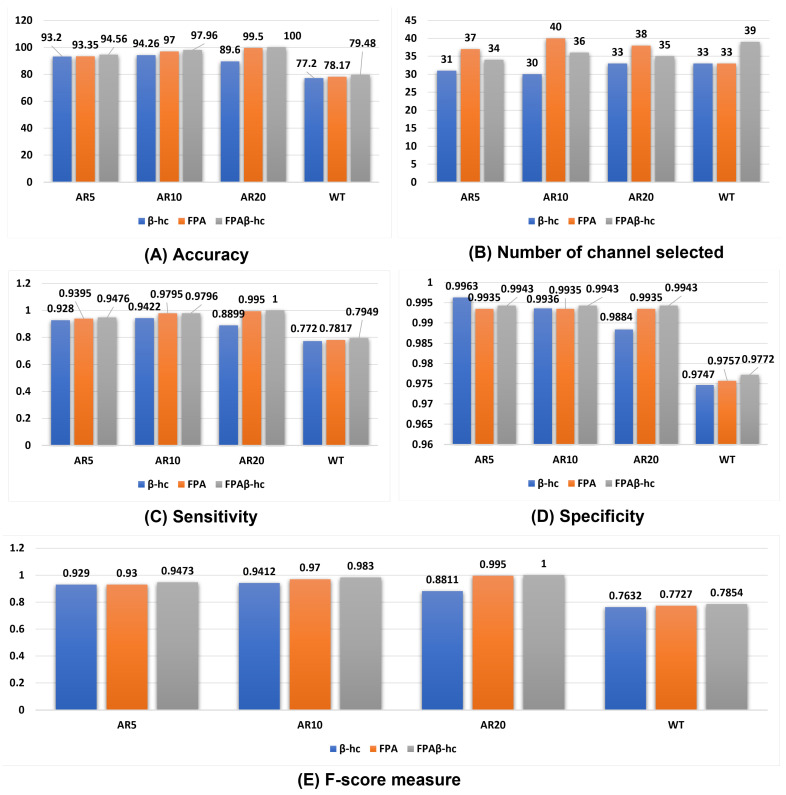
Performance results of the proposed approach over different feature extraction methods.

The proposed approach provides an excellent compromise between the number of selected channels and the remaining evaluation measurements compared to β-hc. Figure 6 depicts the results considering all measures. Additionally, we conducted the Wilcoxon signed-rank statistical test to check if there is a substantial variance between standard FPA and FPAβ-hc. The statistical outcomes are tabulated in Table 7 in terms of P-value, w-value, z-value, and T-Sig. For all experiments (i.e., AR models and WT), FPAβ-hc results are significantly better than basic FPA. The proposed FPAβ-hc-SVM obtained success in achieving the best results compared with standard FPA and β-hc EEG channel selection. This points out the power of hybridization to complement their advantage and avoid their shortcomings (i.e., FPA and β-hc).

### 3.3. Comparison with State-of-the-Art

**Figure 7:**
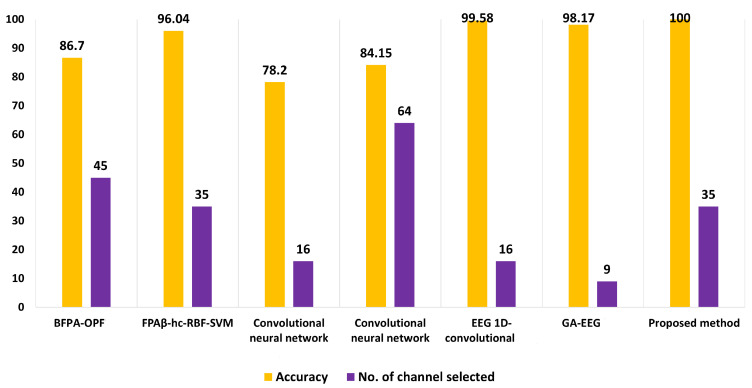
Comparison of the proposed approach with state-of-art methods.

The proposed FPAβ-hc-SVM-RBF is compared against six different state-of-the-art approaches. These methods were carefully selected from the literature, where some are used as a metaheuristic algorithm to select the optimal number of channels: Binary Flower pollination Algorithm with OPF classifier (BFPA-OPF) [1], hybrid FPA with β-hill climbing [2], Genetic algorithm (GA) [26], and a deep learning approach proposed in [27,28]. The comparison involved two criteria, i.e., the accuracy rate and the number of selected channels. The proposed FPAβ-hc showed significant superiority in accuracy criteria, achieving 100% of recognition rate. However, concerning the number of selected, it is ranked third. Figure 7 and Table 8 present a comparison of the accuracy rate using the proposed approach against the methods mentioned earlier.

## 4. Discussions

As aforementioned, the primary purpose of this study is to evaluate the proposed FPAβ-hc-SVM for EEG-based user identification. In this work, we modeled the channel selection task as an optimization problem and introduced the SVM classifier for EEG-based biometric user identification. One can observe the proposed methods achieved similar accuracy rates using SVM considering three different autoregressive coefficients and wavelet features, with an advantage to FPAβ-hc-SVM when compared to standard FPA and β-hc optimizers.

Concerning the number of selected channels, FPAβ-hc-SVM has succeeded in reducing up to half of the total electrodes. The proposed algorithm reduced the total number of electrodes from 64 to 34, 36, 35, and 39 for $AR_{5}$, $AR_{10}$, $AR_{20}$, and WT, respectively. Moreover, we can observe that different AR coefficients provide different accuracy rates regarding the EEG-based person identification task. The proposed approach obtained 100% of accuracy using only 35 sensors and with $AR_{20}$ features.

Another exciting feature of FPAβ-hc-SVM concerns the location of the selected electrodes. It is worth noticing that the proposed method showed that the most common sensors are located on the frontal, occipital, and parietal lobes, although they also spread along with the head. Such finding is an interesting observation, which means FPAβ-hc-SVM tried to identify channels not too close to each other to obtain relevant details from all over the human brain. Table 6 shows a comparison of the proposed method against some state-of-the-art techniques. It is worth noting that FPAβ-hc-SVM achieved the highest accuracy rate when compared to other methods. However, considering the number of selected channels, some improvements are still needed to achieve a minimum number of channels selected, such as the application of multi-objective optimization.

Compared with the previous work, the proposed approach achieved more accurate results using the same feature extraction method (AR${}_{5}$). The accuracy rate was 93.76%, while the number of channels selected is 32 for the previous work. Here, the proposed method achieved an accuracy rate of 94.56% using 34 channels for the AR${}_{5}$ features. Overall, the proposed method archived the best accuracy results (100%) with only 35 channels, where the previous work achieved the best results with an accuracy of 96 with the same number of selected channels (i.e., 35 channels).

## 5. Conclusions and Future Works

In this work, we proposed a hybrid approach composed of the Flower Pollination Algorithm and the β-hill climbing algorithm (FPAβ-hc-SVM) to address the challenge of channel selection in EEG-based biometric person identification. The hybrid approach between FPA and β-hc algorithm has been designed to improve the local pollination part of the FPA to overcome local minima. It is worth mentioning that another version of hybrid FPA was also introduced in [2]. However, the main differences between the proposed approach and the previous ones are: (i) the hybridization in [2] is used to enhance the quality of the best-achieved solution, but here, the hybridization is used for local pollination solutions; (ii) the feature extraction techniques used in [2] were time domain, frequency domain, and time-frequency domain, and the feature extraction methods used here are computed by wavelet features and auto-regressive features; (iii) furthermore, in [2], the 10-fold cross-validation method is used for the training–testing stage, while in this study, a training–validation–testing stage is used.

The primary purpose of this work is to demonstrate that all electrodes are not needed to achieve a high accuracy rate. Therefore, this paper is introduced to model the problem of channel selection as an optimization problem. The channel’s subset that optimizes the recognition ratio over a validation set is employed as the fitness function.

The proposed approach (FPAβ-hc-SVM) is tested using a standard EEG dataset with 64 EEG channels and the data recorded from 109 individuals. In addition, the performance of the proposed method is evaluated using five criteria, which are (i) Accuracy, (ii) F-Score, (iii) Recall, (v) Specificity, and (iv) the number of the channel selected. The FPAβ-hc-SVM was tested using two different feature extraction methods, i.e., Wavelet feature s(WT) and Auto-regressive models with three different coefficients (i.e., AR${}_{5}$, AR${}_{10}$, and AR${}_{20}$). The outcomes of the experiments presented the introduced method excelled both standard FPA and the one proposed by [1,2,26,27,28]. It is worth noting that, while retaining high accuracy rates, the number of sensors has been lessened by half. Additionally, the outcomes displayed a positive correlation between the number of features obtained from the EEG signal and the accuracy ratio. Such a finding suggests that the proposed approach can remove duplicate and undesirable features while retaining specific features.

On the other hand, the current version of the proposed algorithm has some limitations, which are as follows:

1. The proposed algorithm was tested by splitting EEG datasets into three subgroups, i.e., training, validating, and test sets. This approach may lead to overfitting the results. We recommended trying the FPAβ-hc-SVM using *k*-fold-cross-validation approach instead.
2. The FPAβ-hc-SVM technique was tested using WT features and auto-regressive models only. Future work may recommend testing the proposed method over different features. In addition, we recommend investigating the usage of a multi-objective approach.

## Figures and Tables

**Table 1:** Some relevant works related to EEG channel selection.

Work	Approach	Case Study	Channels	Selected	Accuracy
Rodrigues et al. [1]	Binary Flower Pollination with OPF	Person Identification	64	45	86%
Fraschini et al. [16]	Different connectivity metrics	Person Identification	64	N/a	N/a
Gaur et al. [17]	Person correlation coefficient	Motor Imagery	118	36.58	78.08%
Kaur et al. [11]	Principal Component Analysis	Person Identification	64	64	97.73%
Idowu et al. [18]	Modified Particle Swarm Optimization	Motor Imagery	64	30.4	91.89%
Jayarathne et al. [10]	Common Spatial Patterns	Person Identification	14	14	96.97%

**Table 2:** Parameter setting up.

Algorithm	Parameters
FPA	*p* = 0.8, N = 64, D = 20, and Titr = 100
β-hc	β = 0.5, N = 64, D = 1, and Titr = 100

**Table 3:** Parameter setting up.

Classifier	Parameters
LDA	Preset = Linear, covariance structure = Full
LinearSVM	*C* = 1.00 × 10${}^{11}$, Gamma = 0.01, Kernel = Linear, Standardize data: True
KNN	Kernel = Fine, Distance weight: Equal, Distance: Euclidean, Standardize data: True
ANN	Hidden layer = 32, Learning Rate = 0.3, binary splits = True
Naivebayes	*C* = 0.691, Gamma = 0.95, binary splits = True
J48	confidence factor = 0.25, binary splits = False, seed = 1
OPF	– –
RBF-SVM	*C* = 1.00 × 10${}^{11}$, Gamma = 0.01, Kernel = RBF

**Table 4:** Comparison against FPAβ-hc-SVM-RBF and other classifiers.

Dataset	Measure	FPAβ-hc-SVM-RBF	FPAβ-hc-LSVM	FPAβ-hc-LDA	FPAβ-hc-ANN	FPAβ-hc-NB	β-hc-OPF	FPAβ-hc-J48	FPAβ-hc-KNN
	Acc	**94.5619**	52.66	90.13	35.46	85.20	79.73	80.13	83.06
	No. Ch	**34**	40	40	43	39	39	43	43
AR${}_{5}$	Sen	**0.9476**	0.5266	0.9013	0.3546	0.852	79.73	0.8013	0.8306
	Spe	**0.9943**	0.5704	0.8776	0.2827	0.8772	81.55	0.8351	0.8719
	F-Score	**0.9473**	0.5038	0.8790	0.2765	0.8469	78.61	0.7880	0.8223
	Acc	**97.9619**	50.66	93.46	20.40	76.66	78.53	69.73	80.13
	No. Ch	**36**	39	36	39	37	37	42	45
AR${}_{10}$	Sen	**0.9796**	0.5066	0.9346	0.2040	0.7666	0.7853	69.73	80.13
	Spe	**0.9943**	0.5152	0.9430	0.1744	0.8199	0.8281	0.7068	83.83
	F-Score	**0.983**	0.4661	0.9304	0.1494	0.7574	0.7809	0.6620	79.12
	Acc	**100**	50.66	85.6	20.40	83.40	78.66	76.00	81.46
	No. Ch	**35**	36	36	39	39	41	42	47
AR${}_{20}$	Sen	**1**	0.5066	0.856	0.2040	0.8346	0.7866	0.7600	0.8146
	Spe	**1**	0.5152	0.8822	0.1744	0.8605	0.8214	0.8218	0.8511
	F-Score	**1**	0.4661	0.8505	0.1494	0.8263	0.7775	0.7610	0.8110

**Table 5:** *t*-test result between FPAβ-hc-SVM-RBF and other approaches.

		FPAβ-hc-LSVM	FPAβ-hc-LDA	FPAβ-hc-ANN	FPAβ-hc-NB	β-hc-OPF	FPAβ-hc-J48	FPAβ-hc-KNN
AR${}_{5}$	Mean	0.5266	0.9013	0.3546	0.852	0.8306	0.8013	0.8306
	STD	0.0452	0.0520	0.0652	0.0232	0.0672	0.0774	0.0672
	t-value	44.00	3.9338	43.6743	17.6180	8.1506	8.9398	8.1506
	*p*-value	0.00001	0.000134	0.00001	0.00001	0.00001	0.00001	0.00001
AR${}_{10}$	Mean	0.5066	0.9346	0.204	0.7666	0.7853	0.6973	0.8013
	STD	0.0421	0.0485	0.0483	0.0249	0.0566	0.0711	0.0599
	t-value	41	2.09	62.64	22.46	12.95	16.32	11.27
	*p*-value	0.00001	0.020736	0.00001	0.00001	0.00001	0.00001	0.00001
AR${}_{20}$	Mean	0.5066	0.8560	0.2040	0.8346	0.7866	0.7600	0.8146
	STD	0.0421	0.0579	0.0483	0.0436	0.0461	0.0498	0.0389
	t-value	57.32	12.18	80.62	18.53	22.62	23.56	23.3
	*p*-value	0.00001	0.00001	0.00001	0.00001	0.00001	0.00001	0.00001

**Table 6:** Comparison between FPA, β-hc and FPAβ-hc.

Dataset	Measure	FPA-SVM-RBF	FPAβ-hc-RBF-SVM	β-hc-RBFSVM
	Acc	93.3523	**94.5619**	93.2
	No. Ch	37	34	**31**
AR${}_{5}$	Sen	0.9395	**0.9476**	0.928
	Spe	0.9935	**0.9943**	0.9963
	F-Score	0.93	**0.9473**	0.929
	Acc	97	**97.9619**	94.2667
	No. Ch	40	36	**30**
AR${}_{10}$	Sen	0.9795	**0.9796**	0.9422
	Spe	0.9935	**0.9943**	0.9936
	F-Score	0.97	**0.983**	0.9412
	Acc	99.523	**100**	89.6
	No. Ch	38	35	**33**
AR${}_{20}$	Sen	0.995	**1**	0.8899
	Spe	0.9935	**1**	0.9884
	F-Score	0.995	**1**	0.8811
	EEGAcc	78.1714	**79.48**	77.2
	No. Ch	**33**	39	**33**
WT	Sen	0.7817	**0.7949**	0.772
	Spe	0.9757	**0.9772**	0.9747
	F-Score	0.7727	**0.7854**	0.7632

**Table 7:** Wilcoxon signed-rank test between FPA and FPAβ-hc.

Dataset	*p*-Value	w-Value	z-Value	T-Sig	FPAβhc
AR${}_{5}$	0.05	0	−8.329	0.00058	++
AR${}_{10}$	0.05	72.5	−0.1894	0.008493	++
AR${}_{20}$	0.05	12.5	−2.3062	0.002088	++
WT	0.05	0	−0.14	0.00334	++

++ indicates a significant inclination to FPA*β*-hc.

**Table 8:** Comparison of the proposed method (FPAβ-hc-SVM) with state-of-the-art approaches.

Method	Accuracy (%)	No. Ch	Total Channels No.
BFPA-OPF [1]	86.7	45	64
FPAβ-hc-SVM-RBF [2]	96.04	35	64
Convolutional Neural Network [27]	78.2	16	64
Convolutional Neural Network [27]	84.15	64	64
EEG 1D-convolutional [28]	99.58	16	64
GA [26]	98.17	9	32
Proposed approach	100	35	64

## Data Availability

Not applicable.

## Abbreviations

The following abbreviations are used in this manuscript:

BFPA	Binary Flower Pollination Algorithm
β-hc	β Hill Climbing
EEG	Electroencephalogram
ECoG-BCI	Electrocardiography Brain-Computer Interface
FPA	Flower Pollination Algorithm
RBF	Radial Basis Function
SVM	Support Vector Machines
GA	Genetic Algorithm
GWO	Grey Wolf Optimizer
MRI	Magnetic Resonance Imaging
EA	Evolutionary-based Algorithms
TAs	Trajectory-based Algorithms
SI	Swarm Intelligence

## References

[1] Rodrigues D., Silva G.F., Papa J.P., Marana A.N., Yang X.S. (2016). EEG-based person identification through binary flower pollination algorithm. Expert Syst. Appl..

[2] Alyasseri Z.A.A., Khader A.T., Al-Betar M.A., Alomari O.A. (2020). Person identification using EEG channel selection with hybrid flower pollination algorithm. Pattern Recognit..

[3] Al-Qazzaz N.K., Alyasseri Z.A.A., Abdulkareem K.H., Ali N.S., Al-Mhiqani M.N., Guger C. (2021). EEG feature fusion for motor imagery: A new robust framework towards stroke patients rehabilitation. Comput. Biol. Med..

[4] Alyasseri Z.A.A., Khader A.T., Al-Betar M.A. Electroencephalogram signals denoising using various mother wavelet functions: A comparative analysis. Proceedings of the International Conference on Imaging, Signal Processing and Communication.

[5] Souza L., Oliveira L., Pamplona M., Papa J. (2018). How Far Did We Get in Face Spoofing Detection?. Eng. Appl. Artif. Intell..

[6] Alyasseri Z.A.A., Abasi A.K., Al-Betar M.A., Makhadmeh S.N., Papa J.P., Abdullah S., Khader A.T. (2021). EEG-Based Person Identification Using Multi-Verse Optimizer as Unsupervised Clustering Techniques. Evolutionary Data Clustering: Algorithms and Applications.

[7] Marcel S., Nixon M.S., Li S.Z. (2014). Handbook of Biometric Anti-Spoofing.

[8] Campisi P., La Rocca D. (2014). Brain waves for automatic biometric-based user recognition. IEEE Trans. Inf. Forensics Secur..

[9] Ramadan R.A., Vasilakos A.V. (2017). Brain computer interface: Control signals review. Neurocomputing.

[10] Jayarathne I., Cohen M., Amarakeerthi S. BrainID: Development of an EEG-based biometric authentication system. Proceedings of the 2016 IEEE 7th Annual Information Technology, Electronics and Mobile Communication Conference (IEMCON).

[11] Kaur B., Singh D. Neuro signals: A future biomertic approach towards user identification. Proceedings of the IEEE 2017 7th International Conference on Cloud Computing, Data Science & Engineering-Confluence.

[12] Alyasseri Z.A.A., Khader A.T., Al-Betar M.A., Yang X.S., Mohammed M.A., Abdulkareem K.H., Kadry S., Razzak I. (2022). Multi-objective flower pollination algorithm: A new technique for EEG signal denoising. Neural Comput. Appl..

[13] Alyasseri Z.A.A., Khader A.T., Al-Betar M.A., Papa J.P., Alomari O.A. (2018). EEG Feature Extraction for Person Identification using Wavelet Decomposition and Multi-Objective Flower Pollination Algorithm. IEEE Access.

[14] Abdi Alkareem Alyasseri Z., Alomari O.A., Al-Betar M.A., Awadallah M.A., Hameed Abdulkareem K., Abed Mohammed M., Kadry S., Rajinikanth V., Rho S. (2022). EEG Channel Selection Using Multiobjective Cuckoo Search for Person Identification as Protection System in Healthcare Applications. Comput. Intell. Neurosci..

[15] Alyasseri Z.A.A., Alomari O.A., Makhadmeh S.N., Mirjalili S., Al-Betar M.A., Abdullah S., Ali N.S., Papa J.P., Rodrigues D., Abasi A.K. (2022). EEG Channel Selection for Person Identification Using Binary Grey Wolf Optimizer. IEEE Access.

[16] Fraschini M., Didaci L., Marcialis G.L. (2018). EEG-based personal identification: Comparison of different functional connectivity metrics. bioRxiv.

[17] Gaur P., McCreadie K., Pachori R.B., Wang H., Prasad G. (2021). An automatic subject specific channel selection method for enhancing motor imagery classification in EEG-BCI using correlation. Biomed. Signal Process. Control..

[18] Idowu O.P., Adelopo O., Ilesanmi A.E., Li X., Samuel O.W., Fang P., Li G. (2021). Neuro-evolutionary approach for optimal selection of EEG channels in motor imagery based BCI application. Biomed. Signal Process. Control..

[19] Alyasseri Z.A.A., Khader A.T., Al-Betar M.A., Papa J.P., Alomari O.A., Makhadmeh S.N. (2018). Classification of EEG mental tasks using Multi-Objective Flower Pollination Algorithm for Person Identification. Int. J. Integr. Eng..

[20] Alyasseri Z.A.A., Khader A.T., Al-Betar M.A., Papa J.P., Osama A.A., Makhadme S.N. An efficient optimization technique of EEG decomposition for user authentication system. Proceedings of the 2nd International Conference on BioSignal Analysis, Processing and Systems (ICBAPS).

[21] Alyasseri Z.A.A., Khader A.T., Al-Betar M.A., Papa J.P., ahmad Alomari O. EEG-based person authentication using multi-objective flower pollination algorithm. Proceedings of the 2018 IEEE Congress on Evolutionary Computation (CEC).

[22] Yang X.S. (2012). Flower pollination algorithm for global optimization. International Conference on Unconventional Computing and Natural Computation.

[23] Alyasseri Z.A.A., Al-Betar M.A., Awadallah M.A., Makhadmeh S.N., Abasi A.K., Doush I.A., Alomari O.A. (2021). A Hybrid Flower Pollination with *β*-Hill Climbing Algorithm for Global Optimization. J. King Saud Univ.-Comput. Inf. Sci..

[24] Al-Betar M.A. (2017). *β*-Hill climbing: An exploratory local search. Neural Comput. Appl..

[25] Schalk G., McFarland D.J., Hinterberger T., Birbaumer N., Wolpaw J.R. (2004). BCI2000: A general-purpose brain-computer interface (BCI) system. IEEE Trans. Biomed. Eng..

[26] Albasri A., Abdali-Mohammadi F., Fathi A. (2019). EEG electrode selection for person identification thru a genetic-algorithm method. J. Med. Syst..

[27] Karácsony T., Hansen J.P., Iversen H.K., Puthusserypady S. Brain computer interface for neuro-rehabilitation with deep learning classification and virtual reality feedback. Proceedings of the 10th Augmented Human International Conference 2019.

[28] Sun Y., Lo F.P.W., Lo B. (2019). EEG-based user identification system using 1D-convolutional long short-term memory neural networks. Expert Syst. Appl..

